# Interstitial Lung Disease Outcome Prediction Using Quantitative Densitometry Indices on Baseline Chest Computed Tomography

**DOI:** 10.3390/diagnostics15212665

**Published:** 2025-10-22

**Authors:** Li-Ting Huang, Tang-Hsiu Huang, Chung-Ying Lin, Hao Ho, Yi-Shan Tsai, Chia-Ying Lin, Chien-Kuo Wang

**Affiliations:** 1 Department of Medical Imaging, National Cheng Kung University Hospital, College of Medicine, National Cheng Kung University, Tainan 704, Taiwan; 2 Institute of Clinical Medicine, College of Medicine, National Cheng Kung University, Tainan 701, Taiwan; 3 Division of Chest Medicine, Department of Internal Medicine, National Cheng Kung University Hospital, College of Medicine, National Cheng Kung University, Tainan 704, Taiwan; 4 Institute of Allied Health Sciences, College of Medicine, National Cheng Kung University, Tainan 701, Taiwan; 5 Biostatistics Consulting Center, National Cheng Kung University Hospital, College of Medicine, National Cheng Kung University, Tainan 704, Taiwan; 6 College of Nursing, Kaohsiung Medical University, Kaohsiung 807, Taiwan; 7 Department of Statistics, University of California at Los Angeles, Los Angeles, CA 90095, USA; 8 Clinical Innovation and Research Center, National Cheng Kung University Hospital, Tainan 704, Taiwan

**Keywords:** lung disease, interstitial, multidetector computed tomography, densitometry, prognosis, mortality

## Abstract

**Background/Objectives**: Accurate prognostication for interstitial lung disease (ILD) remains challenging, limiting clinicians’ ability to optimize patient management strategies. This study aimed to evaluate the prognostic value of baseline quantitative CT-derived indices, including total lung volume (TLVcm^3^), normal lung volume% (NLV%), and fibrotic lung volume% (FLV%), for predicting three-year mortality in interstitial lung disease (ILD) patients. **Methods**: A total of 101 ILD patients were retrospectively enrolled. Baseline CT-derived indices, including TLVcm^3^, NLV% (−950 to −700 HU), and FLV% (−600 to +50 HU), were measured on chest CT. Baseline forced vital capacity(FVC)% predicted and diffuse capacity of lungs for carbon monoxide (DLCO)% predicted were collected. Survival analysis used Kaplan–Meier’s curves and log-rank tests. Uni- and multivariate Cox’s proportional hazards regression were performed. Pearson’s correlation was used between CT-derived indices, FVC% predicted, and DLCO% predicted. **Results**: During 3-year follow-up, 30 of 101 patients (29.70%) died. Deceased patients had a significantly lower baseline NLV% (59.27% ± 7.61% vs. 65.02% ± 7.82%, *p* = 0.001) and a higher FLV% (17.64% ± 7.98% vs. 13.34% ± 7.48%, *p* = 0.011) compared with survivors. Multivariate analysis identified baseline NLV% (adjusted hazard ratio 0.88, 95% CI: 0.78–0.99, *p* = 0.034) and DLCO% predicted (adjusted hazard ratio 0.97, 95% CI: 0.95–0.99, *p* = 0.007) as independent predictors of three-year mortality. Patients with NLV% ≤ 64.15 and FLV% ≥ 14.12 showed significantly worse survival outcomes (21.78% vs. 7.92%, *p* < 0.001;19.80% (20/101) vs. 9.90% (10/101), *p* < 0.001). CT-derived indices moderately correlated with FVC% predicted and DLCO% predicted. **Conclusions**: Baseline FLV% ≥ 14.12 and NLV% ≤ 64.15 can effectively stratify and differentiate outcomes in ILD patients. Baseline NLV% has the potential as a prognostic indicator for 3-year survival in ILD.

## 1. Introduction

Interstitial lung disease (ILD) encompasses a heterogeneous spectrum of parenchymal lung disorders with overlapping clinical, radiological, and prognostic characteristics [1]. Variable scarring of the interstitium and alveoli after lung parenchymal inflammation leads to deterioration in lung function [2]. Overall, 30% to 40% of people with ILD develop progressive pulmonary fibrosis, which demonstrates a course toward respiratory failure and confers a median survival of 2.5 to 3.5 years [3]. The incidence of ILD is reported to be increasing worldwide [4]. Among individuals with systemic autoimmune rheumatic diseases, ILD represents both a frequent complication and the leading cause of morbidity and mortality [5].

Forced vital capacity (FVC) and the diffusion capacity of the lungs for carbon monoxide (DLCO) are the two most often used physiological parameters to track disease progression and predict mortality [6]. According to current guidelines [6,7], high-resolution computed tomography (HRCT) is the gold standard for the early detection of ILD and allows non-invasive disease extent and morphology evaluation. More extensive fibrosis or ground glass opacities seen in follow-up HRCT indicate radiological evidence of disease progression [6,8,9]. However, the visual assessment of HRCT is limited by high inter-observer variability, reproducibility, and high time requirements [10]. To overcome these shortcomings, the quantitative analysis of HRCT has been extensively studied. Quantitative computed tomographic densitometry, such as mean lung density, skewness, and kurtosis, has yielded reproducible results corresponding to physiological impairment in ILD [11]. However, whole-lung analytical approaches faced challenges in characterizing discrete pathological patterns [12], particularly in ILD, which is characterized by spatially heterogeneous lesions that manifest concurrently within the same anatomical region. To overcome these inadequacies, focal lung histograms for quantifying the area percentage of focal lung lesions using predetermined density ranges [11] and machine learning-based methods employing feature analysis [13] have been investigated.

In this study, we quantitatively measured baseline CT-derived indices, including total lung volume (TLVcm^3^), normal lung volume% (NLV%), and fibrotic lung volume% (FLV%), by applying predetermined Hounsfield unit thresholds on chest CT. Our primary objective was to assess the prognostic ability of baseline CT-derived indices, FVC % predicted, DLCO % predicted, and total lung capacity (TLC) regarding three-year mortality in ILD patients. Additionally, we analyzed the relationships between these baseline CT-derived indices and pulmonary physiological functions.

## 2. Materials and Methods

### 2.1. Study Population

This retrospective study was conducted at a single university medical center from 1 October 2018 to 31 May 2021, and approved by the Institutional Review Board of National Cheng Kung University Hospital (IRB No. B-ER-111–516, 25 February 2023). The dataset collection and experiments were conducted according to approved ethical guidelines and regulations. The Institutional Review Board granted waived informed consent. Patients meeting ILD diagnostic criteria based on the 2022 ATS/ERS and 2023 ACR/CHEST guidelines [6,7] and multidisciplinary review were enrolled. Inclusion criteria required patients to have a baseline chest CT at the time of ILD diagnosis and pulmonary function tests (PFTs) including FVC% predicted, DLCO% predicted, and total lung capacity % predicted (TLC% predicted) obtained within three months before and after the baseline chest CT. The exclusion criteria were severe motion artifacts on chest CT images, acute pulmonary edema, infectious pneumonia, the presence of primary or secondary lung malignancy at the baseline evaluation, and the absence of baseline PFT data due to patient incapability. Demographic data, including age, sex, and smoking status, were collected. The survival status was evaluated by contacting the patient or retrieving it from medical records. Survival time began from the date of baseline chest CT used for diagnosis. The primary end point was all-cause mortality. Patients who underwent lung transplantation were censored at the date of transplant, and those who were alive or lost to follow-up by the 3-year follow-up were censored at their last recorded visit or by 31 May 2024. Patients were categorized into the surviving and deceased group according to the 3-year follow-up survival status.

### 2.2. CT Image Acquisition

Thoracic CT was performed from the apex of the lung to the diaphragm volumetrically by two multidetector CT scanners (SOMATOM Definition Flash and SOMATOM Definition AS, Seimens Healthineers, Erlangen, Germany). All patients were scanned at the end of full inspiration. The parameters were set at 100 or 120 kVp, 1 or 0.75 mm collimation, an increment of 0.7, and a pitch of 1.05 or 1.1. The axial, coronal, and sagittal thin-slice CT images, set at 2.0 mm slice thickness and 2.0 mm slice interval with sharp kernel (B70f), were reconstructed from images at 0.625 to 1 mm slice thickness. A 512 × 512 image matrix size was obtained. No contrast medium was administrated. 

### 2.3. Quantitative Lung Parenchymal Radiodensity Measurement

Imaging analysis was performed using Aquarius 3D Workstation software version 4.4.13 (TeraRecon Inc., San Mateo, CA, USA). The automatic segmentation of bilateral lungs with the exclusion of major blood vessels and airways up to the subsegmental level, along with the measurement of lung parenchymal radiodensity, was conducted for each CT examination. Post-processed images were evaluated by a cardiothoracic radiologist with 8 years of experience and a radiology technologist with 2 years of experience. The range of Hounsfield units (HU) used to quantify the CT-derived index was defined as below:

(1) Total lung volume (TLVcm^3^): defined as the volume from −1024 HU to −200 HU and automatically segmented by the software.
(2) Normal lung volume % (NLV%): defined as the percentage of lung volume with attenuation between −950 and −700 [14].
(3) Fibrotic lung volume % (FLV%): defined as the percentage of lung volume with attenuation between −600 and +50 HU [15,16].

### 2.4. Statistical Analysis

Data are expressed as the mean ± standard deviation (SD), median with interquartile range (IQR), or number (percentage). Comparisons of parameters between the surviving and deceased group were performed. Student’s *t*-test or the Mann–Whitney U test was used for continuous variables with normal or non-normal distributions, respectively. The *X*^2^ test and Fisher’s exact tests were used for categorical variables. Pearson’s correlation was used to evaluate the correlation between the baseline CT-derived index and pulmonary function test (TLC % predicted, FVC % predicted, and DLCO % predicted). The strength of correlations in absolute values was defined as follows [17]: (1) r > 0.90: very strong relationship, (2) r >0.70 to 0.89: strong relationship, (3) r > 0.40 to 0.69: moderate relationship, (4) r > 0.10 to 0.39: weak relationship, (5) r ≤ 0.10: negligible relationship. Receiver operating characteristic (ROC) was used to evaluate the optimal cut-off value of CT-derived indices for 3-year mortality in the study cohort. Kaplan–Meier’s survival curves and log-rank tests were used to investigate the time from diagnosis to death, transplant, loss to follow-up, or the end of the study period (i.e., follow-up duration). Univariate Cox’s proportional hazard models were used to examine the association between selected variables and ILD survival. Bonferroni’s correction was applied to adjust for multiple comparisons. Variables with statistical significance (adjusted *p* < 0.05) in the univariate analysis, along with variables considered clinically relevant (age, gender, and smoking history), were further included in the multivariate model to identify independent predictors of mortality among the ILD patients. *p* < 0.05 was considered statistically significant, and 95% confidence intervals were used to report the precision of our results. SPSS (Statistical Package for the Social Sciences) version 17 software (SPSS Inc., Chicago, IL, USA) was used for all analyses.

## 3. Results

### 3.1. Patient Characteristics

**Figure 1:**
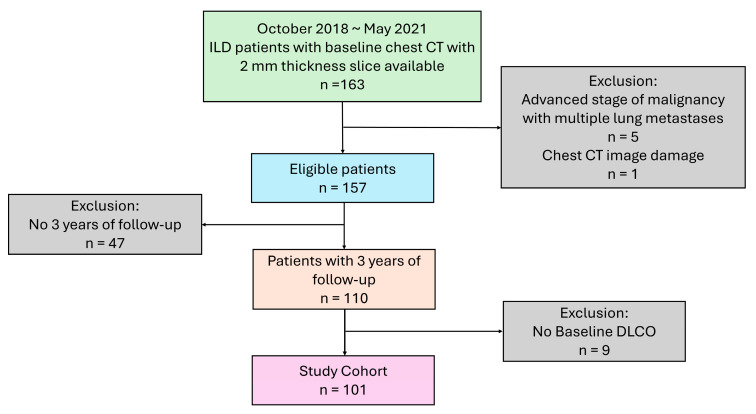
Flow chart of study participants.

A total of 101 patients with a baseline CT at diagnosis and PFT acquired nearest to the baseline CT were enrolled in the study cohort (Figure 1). In the study cohort, the mean age (SD) was 69.41 (12.26). Over the study period, 30 (29.70%) patients passed away. The causes of death were the acute exacerbation of ILD (*n* = 5), infection pneumonia (*n* = 4), the progression of ILD (*n* = 10), acute respiratory failure (*n* = 6), acute hepatitis complicated with acute liver failure (*n* = 1), acute myocardial infarction complicated with acute heart failure (*n* = 1), and sudden cardiac arrest (*n* = 3). The median (IQR) interval times of the baseline HRCT between baseline FVC % predicted, DLCO % predicted, and TLC% predicted were 0.40 (0.00, 1.87), 2.28 (0.13, 5.79), and 0.60 (0.00, 2.50) months, respectively. The demographic data is summarized in Table 1.

### 3.2. Correlations of Baseline PFT and CT-Derived Indices

**Figure 2:**
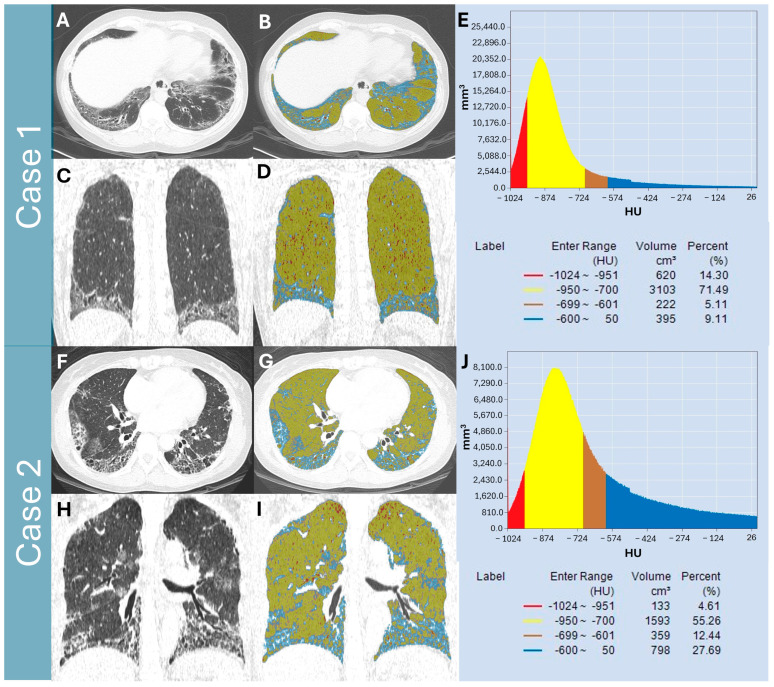
**Two representative cases of quantitative lung parenchymal radiodensity measurement.** Representative focal histograms demonstrating quantitative CT analysis using predetermined Hounsfield unit (HU) thresholds. Normal lung volume (NLV%, yellow) was defined as regions with densities between −950 HU and −700 HU. Fibrotic lung volume (FLV%) was calculated as the density ranges from −600 HU to +50 HU (blue). Case 1: nonspecific interstitial pneumonia in a 46-year-old female diagnosed with primary Sjogren syndrome. The high-resolution CT axial image (**A**) and coronal image (**B**) reveal a mixture of ground glass opacity (GGO) and reticulation. The color-overlayed CT axial image (**C**) and coronal image (**D**) provide visual assessment of lung lesions. The histogram (**E**) showed the quantitative results for each CT-derived index. Case 2: nonspecific interstitial pneumonia in a 50-year-old female diagnosed with dermatomyositis. The CT axial image (**F**) and coronal image (**G**) reveal GGO, fine mixed coarse reticulations, and focal fibrotic consolidation. The color-overlay axial image (**H**) and coronal image (**I**) demonstrated the corresponding lesions. The histogram (**J**) showed the quantitative results for each CT-derived index.

**Figure 3:**
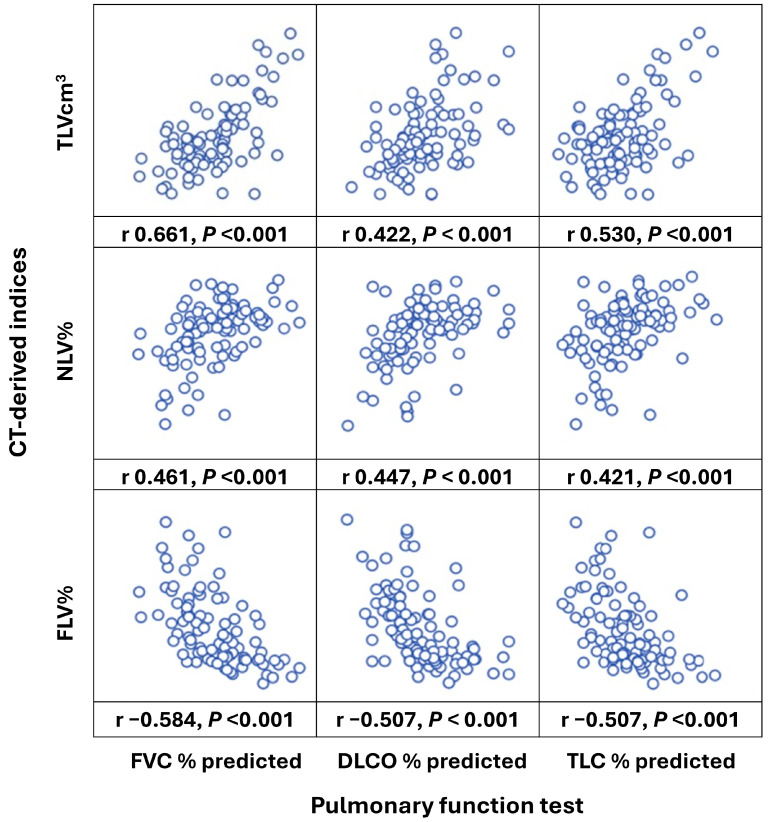
**Pearson’s correlation for baseline CT-derived indices and pulmonary function test.** Pearson’s coefficient (r) with *p* value is shown below each scatter plot. TLC % predicted, predicted percentage of total lung capacity; DLCO % predicted, predicted percentage of diffusing capacity of lungs for carbon monoxide; FVC % predicted, predicted percentage of forced vital capacity; TLVcm^3^, total lung volume (cm^3^); NLV%, normal lung volume%; FLV%, fibrotic lung volume %.

The representative figures for quantitative lung parenchymal radiodensity measurement are shown in Figure 2. In 101 patients, there was a moderate, negative correlation observed between FLV% and FVC % predicted (r = −0.584, *p* < 0.001), DLCO % predicted (r = −0.507, *p* < 0.001), and TLC % predicted (r = −0.507, *p* < 0.001). Moderate positive correlations were found between NLV% and FVC % predicted (r = 0.461, *p* < 0.001), DLCO % predicted (r = 0.447, *p* < 0.001), and TLC % predicted (r = 0.421, *p* < 0.001). In addition, there were moderate positive correlations between TLVcm^3^ and FVC % predicted (r = 0.661, *p* < 0.001), DLCO % predicted (r = 0.422, *p* < 0.001), and TLC % predicted (r = 0.530, *p* < 0.001). The correlation summary of the PFT and CT-derived indices is presented in Figure 3.

### 3.3. Comparison Analysis Between Surviving and Deceased Patients

**Figure 4:**
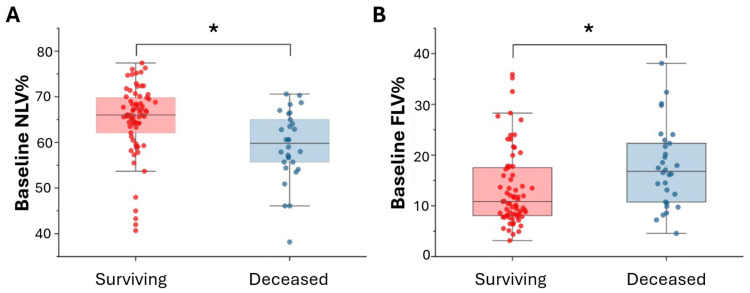
Baseline NLV% and FLV% comparison between the surviving and deceased patients. (**A**) The baseline NLV% was significantly higher in the surviving group compared with the deceased group (65.02% ± 7.82% vs. 59.27% ± 7.61%, *p* = 0.001). (**B**) The baseline FLV% was significantly elevated in the deceased group compared with the surviving group (17.64% ± 7.98% vs. 13.34% ± 7.48%, *p* = 0.011). *: *p* < 0.05.

In the study cohort, there were significant increases in baseline NLV% in the surviving patients compared with the deceased patients (65.02% ± 7.82% vs. 59.27% ± 7.61%, *p* = 0.001, Figure 4A). The FLV% was significantly higher in the deceased patients compared with the surviving patients (17.64% ± 7.98% vs. 13.34% ± 7.48%, *p* = 0.011, Figure 4B). There was no significant difference in baseline TLVcm^3^ between the surviving and deceased patients (3223.55 cm^3^ ± 941.68 cm^3^ vs. 2969.87 cm^3^ ± 859.48 cm^3^, *p* = 0.208). The deceased patients had significantly lower baseline FVC % predicted and DLCO% predicted compared with the surviving patients (68.43% ± 18.95% vs. 79.34% ± 16.05%, *p* = 0.004; 53.13% ± 22.99% vs. 68.87% ± 22.37%, *p* = 0.002, respectively). TLC % predicted was not significantly different between the surviving and deceased patients (78.33% ± 15.87% vs. 71.52% ± 17.51%, *p* = 0.063). The comparison results are demonstrated in Table 2. 

### 3.4. Mortality Prediction of Baseline CT-Derived Indices

**Figure 5:**
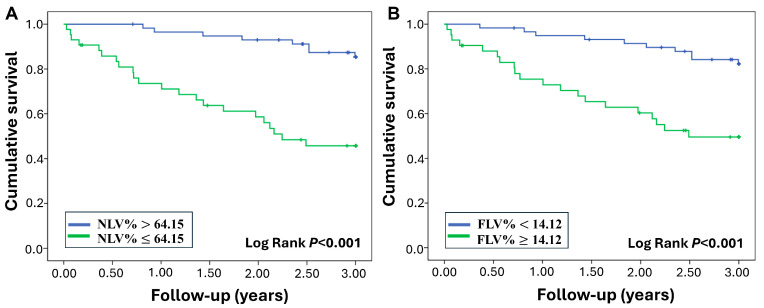
**Comparison of survival outcomes. Kaplan–Meier’s survival curves for** (**A**) **NLV%,** (**B**) **FLV%**. Patients with NLV% ≤ 64.15 showed higher mortality than those with NLV% > 64.15 (3-year mortality: 21.78% (22/101) vs. 7.92% (8/101), log-rank *p* < 0.001). Patients with FLV% ≥ 14.12 exhibited higher mortality than those with FLV% < 14.12 (3-year mortality: 19.80% (20/101) vs. 9.90% (10/101), log-rank *p* < 0.001). NLV%, normal lung volume %; FLV%, fibrotic lung volume %.

The univariate Cox regression analysis revealed that baseline FVC% predicted, DLCO% predicted, NLV%, and FLV% are statistically significant predictors of mortality in ILD (Table 3). The multivariate Cox regression analysis resulted in a model that found baseline NLV% (adjusted hazard ratio 0.88, 95% CI: 0.78–0.99, *p* = 0.034) and baseline DLCO% predicted (adjusted hazard ratio 0.97, 95% CI: 0.95–0.99, *p* = 0.007) to be significant predictors for survival (Table 4). The ROC analysis yielded optimal threshold values for NLV% at 64.15%, demonstrating a sensitivity of 77.33% and a specificity of 70.42%. For FLV%, the optimal cut-off value was established at 14.12%, achieving a sensitivity of 66.67% and a specificity of 69.01%. During the 3-year follow-up, patients with NLV% ≤ 64.15 had significantly worse survival than those with NLV% > 64.15 (3-year mortality: 21.78% (22/101) vs. 7.92% (8/101), log-rank *p* < 0.001) (Figure 5A). Those who were presented with FLV% ≥ 14.12 showed significantly higher mortality than those with FLV% < 14.12 (3-year mortality: 19.80% (20/101) vs. 9.90% (10/101), log-rank *p* < 0.001) (Figure 5B).

## 4. Discussion

The present study demonstrated that baseline NLV% and DLCO% predicted were two independent prognostic factors for 3-year mortality after adjustment for sex, age, smoking status, baseline FVC% predicted, and baseline FLV%. Baseline NLV% and DLCO% predicted were associated with an 0.88-fold and 0.97-fold increased hazard of 3-year mortality, respectively. The baseline CT-derived indices showed a good correlation with lung physiology parameters among patients with ILD. Compared with surviving patients, deceased patients had a significantly higher baseline FLV% and significantly reduced NLV%.

ILD encompasses a broad spectrum of subtypes and shows overlapping imaging features on CT, including ground glass opacity, reticulations, honeycombing, and focal airspace consolidation with/without decreased lung volume [18]. In previous studies, lesions with a density between −600 HU and −250 HU have been recognized for primary reticulation and ground glass opacity (GGO) [19] and extracellular matrix remodeling [20]. Lung parenchymal disease patterns with a density ranging from −249 HU to +50 HU were reported as focal airspace opacity associated with a fibrotic background or coarse reticulations [16]. In this study, we measured the fibrotic lung volume with lung parenchymal density between −600 HU and +50 HU. We observed that the presence of fibrotic consolidation, coarse reticulations, thick wall honeycombing cysts, and subpleural linear densities showed dominant densities between 249 HU to +50 HU. Applying the threshold between −600 HU and +50 HU enhanced the coverage of ILD lesions. This findings aligns with recent reports suggesting that the inclusion of coarse reticulations enhances the sensitivity for detecting disease progression and showed potential in identifying progressive ILD from non-progressive ILD [21]. Various histogram-based quantitative CT for quantifying fibrosis burden in ILD have been tested due to the absence of a standardized consensus. Despite the use of different threshold ranges, significant negative correlations with DLCO and FVC% have been consistently reported [22,23]. The negative correlations between FLV% and both FVC% predicted and DLCO% predicted indicate a higher percentage of coarse reticulation, focal fibrotic consolidation, subpleural linear densities, and thicker or increased honeycombing cysts, all of which affect lower pulmonary physiological function. In addition, FLV% was significantly higher in deceased patients at baseline. Therefore, FLV% on baseline CT could be a reasonable imaging biomarker to cover the lesion spectrum from fibrosis and organizing lung injury and correlate with physiological impairment in ILD patients. The CT value of −950 HU is currently the most accepted demarcation between emphysema and normal lungs. The threshold has been validated through accurate correlation with histologically defined emphysema [24]. In contrast, the CT threshold that distinguishes normal lungs from interstitial lung disease varies across studies, primarily due to different disease cohorts [25]. We followed the previously proposed threshold applied for patients with interstitial lung disease [14,26,27] by defining the percentage of lung area between −700 and −950 HU for NLV%. The NLV% showed moderately positive correlations with FVC% predicted and DLCO% predicted, compatible with a previous study on diffuse interstitial lung disease [27]. Furthermore, we found that the baseline NLV% was significantly lower in deceased patients compared with surviving patients. This suggests that NLV% could be a useful parameter for evaluating lung physiological function.

The correlation between CT-derived TLVcm^3^ and TLC was comparable to a previous study [27]. CT-derived TLVcm^3^ can be effectively obtained using clinical scan protocols on multiple types of CT scanners with various reconstruction techniques, independent of specific lung diagnoses, age, height, or body mass index [28,29]. Although plethysmography has been regarded as the gold standard for TLV measurement [30], it requires skilled technicians and specialized expensive equipment to perform qualified examinations. Chest CT is conducted following routine preparation and standard instructions. It can provide various information on interstitial lung abnormalities and total lung volume in one session without further expenditure. However, a supine position causes an underestimation of actual lung volume with CT compared with plethysmography [31]. The breathing protocols also differ between spirometry and chest CT. Consequently, CT-derived TLVcm^3^ cannot be directly equated to TLC but can be an alternative when patients are unable to tolerate plethysmography [28].

We followed newly diagnosed ILD patients for at least 3 years from the time of diagnosis. This is compatible with previous studies, which had a follow-up duration of 2 to 3 years [32,33]. Baseline NLV% and FLV% could potentially be useful factors in stratifying mortality risk in ILD patients. Patients with NLV% ≤ 64.15 showed significantly worse survival outcomes, suggesting that this threshold could be valuable for risk stratification. Similarly, FLV% ≥ 14.12 was associated with higher mortality, highlighting the prognostic importance of fibrotic changes visible on CT imaging. Previous studies employing quantitative CT biomarkers have primarily demonstrated the associations with ILD outcomes [23,34]; this study proposes baseline CT-derived volumetric indices with explicit thresholds for 3-year mortality risk stratification in a heterogeneous ILD cohort. 

In univariate regression analysis, FVC % predicted and DLCO % predicted are significant factors for mortality. These findings align with the previously established literature, wherein FVC and DLCO are the most common PFT parameters associated with ILD prognosis [6]. Notably, in multivariate analysis, both baseline NLV% and DLCO % predicted emerged as independent predictors of 3-year mortality. This finding suggests that the integration of quantitative CT measurements with traditional pulmonary function testing could enhance risk stratification in ILD patients. The complementary nature of these parameters—with NLV% providing the direct visualization of preserved lung tissue and DLCO% reflecting gas exchange capacity—offers clinicians a more nuanced assessment of disease severity and prognosis.

Recent advances in machine learning have improved the efficiency, precision, and reproducibility of segmentation, registration, and pattern classification [35]. AI-based ILD volume percentage was strongly correlated with functional impairment and reported as an independent prognostic predictor [22,36,37,38,39]. In addition, AI-based models were investigated to integrate longitudinal data and dynamically predict 3-year survival in ILD [40]. However, because the quality of labeled training data is highly dependent on expert radiologists, the standardization of data labeling is still challenging due to the unsatisfactory inter-observer agreement [41]. In addition, the explainability of model results remains an unresolved issue, especially when there is a discrepancy between expert opinion and output from deep learning algorithms, as well as the awareness of the misclassification of images by software. At present, commercialized machine learning-based algorithms for quantifying ILD on CT are not widely available, and the lack of head-to-head comparisons limits their deployment in clinical practice [23]. In contrast, our study had several advantages. The radiodensity-based measurement employed in this study provides a straightforward, intuitive, and automated approach to disease assessment. This technique enabled the objective, visualizable, and reproducible quantification of disease extent. High-resolution volumetric CT images of the entire lung using a 2 mm slice thickness can be acquired using standardized protocols during routine clinical scanning and requiring no additional preparation. The method used in this study provides a simple approach that can be seamlessly integrated into routine clinical workflows. 

The study has several limitations. First, our sample size was relatively small, and an independent validation cohort was lacking. This restricts the generalizability of our findings and may introduce selection bias inherent to the retrospective, single-center design. Future multicenter, prospective studies with larger sample sizes are needed to validate these findings and establish the clinical utility of radiodensity-based measurements in ILD prognosis. Second, the limited sample size precluded subgroup analysis comparing patients with and without antifibrotic treatment. Future larger-scale studies should incorporate stratified analyses combining CT-derived indices with serum protein, autoantibodies associated with ILD development, and FVC to better evaluate treatment response and disease progression [42]. Third, our analysis revealed certain technical limitations in the automated segmentation process. Small central areas within honeycombing cysts were occasionally misclassified as NLV%, while some peripheral perivascular regions were incorrectly calculated as FLV%. These errors arise from the algorithm’s dependence on attenuation difference between parenchymal structures, which become less distinct in smaller structures due to spatial resolution constraints and partial volume averaging [43]. While multidetector CT acquisition with thin, contiguous slices can mitigate partial volume effects, the manual correction of these segmentation errors remains resource-intensive and impractical for routine clinical workflows. Recent advances in machine learning techniques offer promising solutions for more accurate and efficient lung lesion segmentation, potentially overcoming these current technical challenges [39].In conclusion, this study demonstrated that quantitative analysis on baseline chest CT is valuable for prognostic assessment in ILD. Baseline CT-derived indices, including NLV% ≤64.15% and FLV% ≥ 14.12%, were associated with risk stratification and multivariate analysis identified baseline NLV% and DLCO% predicted as independent predictors of 3-year mortality. These findings highlighted the complementary roles of quantitative CT metrics alongside pulmonary function testing in prognostic evaluation. The correlation between CT indices and PFT parameters suggests that quantitative CT analysis might be particularly valuable in cases where PFT cannot be performed reliably. Future multicenter, prospective studies with larger cohorts and external validation are needed to confirm these thresholds of CT-derived indices and establish their utility in individualized risk stratification and the monitoring of treatment response. 

## Figures and Tables

**Table 1:** Demographic characteristics of study subjects.

	All Patients
Characteristics	(*n* = 101)
**Demographics**	
Females	38 (37.62%)
Age (years)	69.41 ± 12.26
Smoking status	
Never	59 (58.42%)
Ever	42 (41.58%)
**Baseline pulmonary function**	
FVC (% predicted)	76.10 ± 17.60
DLCO (% predicted)	64.20 ± 23.58
TLC (% predicted)	76.32 ± 16.58
TLC (L)	3.58 ± 0.97
**Baseline CT-derived index**	
Total lung volume (cm^3^)	3148.20 ± 921.16
Normal lung volume (%)	63.31 ± 8.15
Fibrotic lung volume (%)	14.62 ± 7.85
**Outcome**	
Surviving	71 (70.30%)
Deceased	30 (29.70%)
**Follow up (years)**	3.00 (1.99, 3.00)

**Table 2:** Comparison of characteristics between survivors and the deceased in the 3-year follow-up.

	Surviving	Deceased	
	(*n* = 71)	(*n* = 30)	*p*
**Age**	67.93 ± 12.80	72.87 ± 10.24	0.065
**Gender**			
Female	30 (40.25%)	8 (26.67%)	0.140
Male	41 (57.75%)	22 (73.33%)	
**Smoking status**			
Never smoked	41 (57.75%)	18 (60.00%)	0.834
**Baseline FVC % predicted**	79.34 ± 16.05	68.43 ± 18.95	0.004
**Baseline DLCO % predicted**	68.87 ± 22.37	53.13 ± 22.99	0.002
**Baseline TLV% predicted**	78.33 ± 15.87	71.52 ± 17.51	0.063
**Baseline TLV (L)**	3.66 ± 0.94	3.39 ± 1.03	0.217
**Baseline CT-derive index**			
TLVcm^3^	3223.55 ± 941.68	2969.87 ± 859.48	0.208
NLV%	65.02 ± 7.82	59.27 ± 7.61	0.001
FLV%	13.34 ± 7.48	17.64 ± 7.98	0.011

**Table 3:** Univariate Cox’s regression analysis for risk factors of 3-year mortality.

Variables	HR	95% CI	*Raw p*	*Adjusted p*
Female	−			
Male	1.74	0.78–3.91	0.179	1.432
Age	1.03	0.10–1.06	0.079	0.632
Never smoker	−			
Ever smoker	0.97	0.47–2.01	0.932	7.456
Baseline DLCO% predicted	0.97	0.95–0.99	0.000	0.000
Baseline FVC% predicted	0.96	0.94–0.99	0.001	0.008
Baseline TLVcm^3^	1.00	1.00–1.00	0.205	1.640
Baseline NLA%	0.94	0.91–0.97	0.000	0.000
Baseline FLA%	1.06	1.02–1.10	0.005	0.040

**Table 4:** Multivariate Cox regression analysis for risk factors of 3-year mortality.

Variables	HR	95% CI	*p*
Female	−		
Male	2.70	0.92–7.91	0.070
Age	1.02	0.97–1.06	0.480
Never smoker	−		
Ever smoker	0.64	0.26–1.56	0.326
Baseline FVC	0.98	0.95–1.01	0.229
Baseline DLCO	0.97	0.95–0.99	0.007
Baseline NLA%	0.88	0.78–0.99	0.034
Baseline FLA%	0.90	0.79–1.02	0.103

## Data Availability

The original contributions presented in this study are included in the article. Further inquiries can be directed to the corresponding author.

## Abbreviations

The following abbreviations are used in this manuscript:

ILD	interstitial lung disease
GGO	ground glass opacity
TLVcm^3^	total lung volume
NLV%	normal lung volume %
FLV%	fibrotic lung volume %
PFT	pulmonary function test
FVC	forced vital capacity
DLCO	diffusing capacity of lungs for carbon monoxide
IQR	interquartile range
